# Supplementary material for: Loss of Heterozygosity in the Tumor DNA of De Novo Diagnosed Patients Is Associated with Poor Outcome for B-ALL but Not for T-ALL
Source: Genes (Basel). 2022 Feb 23;13(3):398. doi: 10.3390/genes13030398 (PMC8952291; doi:10.3390/genes13030398)
Supplement: Supplementary file 1 [file genes-13-00398-s001.zip › Table S1.pdf]

Table S1. Distribution of LOH by loci in B- ALL LOH-positive patients.

Patient`s [illegible]	ALL phenotype	D1S1656	D2S441	D3S1358	D5S818	D7S820	D8S1179	D10S1248	D12S391	D13S317	D16S539	D18S51	D21S11	D22S1045	CSF1PO	FGA	SE33	TH01	TPOX	VWA	Amelogenin X	Amelogenin Y	Karyotype
		1q42	2p14	3p21.31	5q23.2	7q21.11	8q24.13	10q26.3	12p13.2	13q31.1	16q24.1	18q21.33	21q21.1	22q12.3	5q33.1	4q31.3	6q14	11p15.5	2p25.3	12p13.31	Xp22.1 –[illegible]	Yp11.2	
29	B-II	LOH								LOH							LOH						A
32	B-II			LOH																			A
39	B-II				LOH	LOH					LOH				LOH	LOH							N
45	B-II	LOH						LOH				LOH				LOH	LOH					LOH	A
63	B-II		H		LOH			H	H	H	H					H			H				N
65	B-II		H						LOH					H				H		LOH			A
89	B-IV	LOH	H						LOH		LOH			H	H				H	H			A
71	B-II		H		H		LOH	LOH			H	LOH		H	H	LOH	LOH			H		LOH	A
82	B-II			H						H	H						LOH						A
91	B-II	LOH	H								H				LOH			H		H			A
	Total LOH	4	0	1	2	1	1	2	2	1	2	2	0	0	2	3	4	0	0	1	0	2	
	Total LOH, % of 40 B-ALL patients	10%	0%	2,5%	5%	2,5%	2,5%	5%	5%	2,5%	5%	5%	0%	0%	5%	7,5%	10%	0%	0%	2,5%	0%	5%	

*H - homozygous locus, N - normal karyotype, A - abnormal karyotype
