# Supplementary material for: Loss of Heterozygosity in the Tumor DNA of De Novo Diagnosed Patients Is Associated with Poor Outcome for B-ALL but Not for T-ALL
Source: Genes (Basel). 2022 Feb 23;13(3):398. doi: 10.3390/genes13030398 (PMC8952291; doi:10.3390/genes13030398)
Supplement: Supplementary file 1 [file genes-13-00398-s001.zip › Table S2.pdf]

Table S2. Distribution of LOH by loci in B- ALL LOH-positive patients.

Patient`s [illegible]	ALL phenotype	D1S1656	D2S441	D3S1358	D5S818	D7S820	D8S1179	D10S1248	D12S391	D13S317	D16S539	D18S51	D21S11	D22S1045	CSF1PO	FGA	SE33	TH01	TPOX	VWA	Amelogenin X	Amelogenin Y	Karyotype
		1q42	2p14	3p21.31	5q23.2	7q21.11	8q24.13	10q26.3	12p13.2	13q31.1	16q24.1	18q21.33	21q21.1	22q12.3	5q33.1	4q31.3	6q14	11p15.5	2p25.3	12p13.31	Xp22.1_22.3	Yp11.2	
2	T-III							LOH															N
3	T-II								LOH											LOH			A
4	T-III								LOH														A
33	T-III								LOH							LOH				LOH			A
43	T-III								LOH											H			A
64	T-III							H	H	H	H		LOH										N
66	T-III	H					H	LOH		H													N
85	T-III		H		H	H					H				H			LOH					N
88	T-III			H		H	H					H					LOH	H					A
	Total LOH	0	0	0	0	0	0	2	4	0	0	0	1	0	0	1	1	1	0	2	0	0	
	Total LOH, % of 44 T-ALL patients	0%	0%	0%	0%	0%	0%	4,6%	9,1%	0%	0%	0%	2,3%	0%	0%	2,3%	2,3%	2,3%	0%	4,5%	0%	0%	

*H - homozygous locus, N - normal karyotype, A - abnormal karyotype
